# An interactive simulator for integrating biochemical models with experimental data

**Published:** 2026-09-28

**Authors:** Herbert M. Sauro, Dan Nguyen, Long Nguyenle, Steven S. Andrews, Frank T. Bergmann, Joseph L. Hellerstein, Lucian P. Smith, H. Steven Wiley

**Affiliations:** 1 Department of Bioengineering, University of Washington, Seattle, 98195-5061, WA, USA; 2 Biological Sciences Division, Pacific Northwest National Laboratory, 902 Battelle Blvd, Richland, 99354, WA, USA; 3 eScience Institute, University of Washington, Seattle, 98195-5061, WA, USA; 4 BioQUANT/COS, Heidelberg University, INF 267, 69120 Heidelberg, Germany; 5 Allen School of Computer Science, University of Washington, Seattle, 98195-5061, WA, USA

## Abstract

Biological models based on mathematical simulations (e.g., ordinary differential equations) are hugely beneficial for understanding biological processes and predicting the outcomes of experiments. Such models are built using a variety of sophisticated modeling tools. Unfortunately, the complexity of these tools is often a barrier to use by experimental biologists. To improve the usability and impact of biological models, we have created a desktop platform (DeskIridium) and a web-based simulator (WebIridium, hosted as a GitHub page) that, in combination, provide features that promote ease of use by experimental biologists, including: human-readable editing of models via the Antimony syntax; sliders for adjusting parameter values while simultaneously displaying simulation results in real time; zero-install distribution; AI chat integration; and generation of standalone Python implementations of SBML models to support reproducibility. The desktop version uses the well-established libroadrunner simulation package; the web-based application uses an Emscripten translated version of COPASI. Both simulators are SBML-compatible, allowing users to easily import previously built models or edit existing models using the easy-to-use antimony language. We used modern AI methods for software development and share the lessons we learned. Binaries, source code, and the web interface are available at: https://github.com/sys-bio/IridiumSimulator and https://github.com/sys-bio/WebIridium.

## Introduction

Biochemical systems are typically too complicated to understand without the aid of simulation tools. As a result, these tools have become essential to modern biological research. The earliest is from 1943 when Briton Chance used the mechanical differential analyzer to model the action of the enzyme peroxidase [1]. In 1952 Chance and coworkers used a custom-built electronic analog computer to model the action of catalase [2]. From the late 1950s onward, however, the advent of the digital computer with its high speed and capacity coupled to potentially much greater numerical precision soon displaced the analog computer as the preferred medium, although analog computers were still used in the 1960s [3, 4]. By the 1980s the personal computer became a viable platform for running simulations [5] and today, we have access to unprecedented computer power as well as rich user interface and graphical capabilities. In 2003, the first exchange format, SBML [6], for biochemical models was released. This resulted in the emergence of large repositories of models such as BioModels [7] where models are easily exchangeable and reusable. Biochemical simulation applications are now commonplace and are available both as graphical user interfaces [8, 9] and as packages for scripting languages such as Python [10–12].

This paper introduces the Iridium simulation platform, a desktop simulator (DeskIridium) and a web-based simulator (WebIridium). This platform is specifically geared toward ease of use and, uniquely, rapid interactive operation. The objective in designing Iridium was to make computer simulation painless and productive. Many of the software tools available today require, in some cases, considerable setup and training. In designing Iridium, we partnered with seasoned modelers who require ease of use and high productivity. This is especially true when models become large.

This article focuses primarily on the novelty of the human interface and the role modern AI can play in software development. For the simulation engines, we use existing tooling. On the desktop, Iridium uses the libroadrunner simulator library [11, 13]. On the Web, we use a Javascript/WASM version of COPASI [8]. The main novel contributions of the Iridium project are:

1. Interactive exploration of parameter values using sliders that provides real-time visualization of simulation results;
2. Exporting models as Python implementations to promote reuse and reproducibility as well as SED-ML and COMBINE archives;
3. A zero-install, universally accessible, and low-maintenance web-based implementation hosted on a static GitHub Page;
4. AI chat that automatically includes model context to provide high-quality AI assistance for modeling, as well as built-in model checking in the desktop version;
5. Fast search of the BioModels repository for models, and fast loading of these models into Iridium.

## 1 Design Decisions

As with all modern simulation platforms, the Iridium platform supports importing and exporting models using SBML. In addition, for reproducibility of simulations and analyses outside the tools, the desktop edition allows users to translate their workflow to an equivalent Python script, that can be run using the Tellurium Python package [12, 14]. The desktop app can also export SED-ML and COMBINE archives.

### 1.1 Guiding Principles

Our guiding philosophy originates from a quote attributed to the computer scientist Alan Key: ‘Simple things should be simple, complex things should be possible’. He coined this expression during his time at Xerox PARC to emphasize that user interfaces and programming languages should be intuitive for beginners yet powerful enough for advanced tasks [15]. Similarly, Larry Wall, the creator of the Perl programming language, expressed a similar sentiment: ‘Easy things should be easy, and hard things should be possible.’ This philosophy guided Perl’s design, aiming to make simple tasks straightforward while ensuring that more complex operations remained achievable [16]. The design of the Iridium platform was driven by these principles.

### 1.2 Panel Structure

The application is a single window (or page on the Web version) that is divided into three panels. From the left, the panels include:

1. Control Panel
2. Input Panel
3. Output Panel

The user can select different tools by switching the control panel via the toolbar. The current range of tools includes the following:

1. Time Course Simulation
2. Steady State
3. Parameter Scan

#### Time Course Panel:

The time course panel can be used to run deterministic simulations of the current model in the input panel. The duration of the simulation and the number of points to generate can be specified, as well as what simulation outputs should be captured. The panel allows a user to specify an independent variable and one or more dependent variables. By default, repeated calls to simulate will reset the initial conditions back to their original values. Alternatively, the panel can be configured so that a new simulation continues from where the last one finished.

All input control panels have a slider option, which, when selected, creates a new slider panel in the central input panel (Figure 1). This allows users to interact with the model in real time. Users can easily create or delete sliders and set their ranges. Users can copy slider positions back to the model if a particular behavior has been identified and needs to be captured in the Antimony script.

#### Steady State Panel:

The steady-state panel is used to compute the steady state of the current model. It will also compute the Jacobian and sensitivity coefficients defined in metabolic control analysis [17, 18], both scaled and unscaled sensitivities. The desktop version also offers a 3D plot of the sensitivities.

Results from the steady state analysis can be copied to the clipboard or saved as a CSV file. As with the time course panel, the steady state panel has a slider option. However, unlike the time course sliders, this does not allow the user to compute the steady state as the slider moves; instead, it computes the steady state only when the user releases the slider. This is because computing the steady state in real time cannot be guaranteed due to the nature of the algorithms employed. Instead, a separate tool has been developed to study how steady state changes as a function of parameters, as well as to provide stability information. This is based on the Julia BifurcationKit package [19] but has been wrapped into an easy-to-use and interactive GUI [20].

#### Parameter Scan Panel:

The parameter scan panel generates data of time courses or specific points in time as a function of changes in a parameter value (Figure 3). Different model outputs can be selected, and one parameter can be selected for a given scan. Scans can be linear, log, or defined by manually assigned values in a list. Scans can be used to track the time course, endpoint, peak value, or time to peak as a function of a given parameter. Scans can also be used to examine the dose response by plotting the response at a given time as a function of dose (Figure 2).

Sliders can also be used with a parameter scan. The only restriction is that the parameter being scanned doesn’t have a slider; only non-scanned parameters have sliders.

#### Input Panel:

The central input panel has two halves. The upper half is an editor where a user can specify a model using the antimony syntax. The editor provides line numbers for error reporting and syntax highlighting.

The lower panel is reserved for other input modalities. In the current version, the only other input mode is the slider panel.

#### Output Panel:

Time course simulation results can be output in two forms: 2D plots or CSV tables. Steady state results are output either as tables or 3D par plots. The 2D plotting control has a dedicated configuration panel that allows users to change colors and other stylistic aspects. All plots can be saved in the form of PDF files, suitable for publication. Bitmap PNG format can also be exported. Alternatively, a user can save the raw data as CSV and use their own plotting software.

Data from an external source, in the form of CSV files, can be overlaid on a simulation study. This allows users to compare a time course simulation with experimental data. Any number of files can be loaded and overlaid or shown individually (Figure 4).

#### Model Checking:

The desktop version has a built-in model checking feature that examines models for errors or warnings such as suspicious rate laws or missing initializations of simulation variables. In WebIridium, the built-in AI assistant provides similar capabilities. The checker will also do dynamic tests and can check and identify, for example, divide-by-zero errors. Model checking is based on a series of eighteen built-in rate-law templates; these can be exported as JSON and modified, or new rate laws can be added by creating new JSON files. A model-checking report is provided as a Markdown document in the reports tab of the output panel. This report, together with the antimony model, can be provided to an AI to fix the identified issues in the model. The appendix contains an example of a report from the checker which has identified a suspicious rate law k1*A/Km and a divide by zero error, since Km = 0.

### 1.3 Simulation Engines

#### Desktop Version

The underlying simulation engine for the desktop version is libroadrunner [11]. This is a high-performance SBML-compliant simulator library with an extensive C and C++ API. The engine was designed to be hostable by other applications, and a variety of tools use it for this purpose [21]. libroadrunner also has an extensive Python interface that allows users to script simulation studies within Python. Iridium uses the C API [22]. to gain access to the libroadrunner’s functionality.

#### Web-based Version

The web-based version uses COPASI [8] as its backend simulator, bringing a robust and SBML-compliant engine directly into the browser. While libRoadRunner is the simulation engine used in the desktop edition, its just-in-time compilation approach is designed for native environments and is not currently compatible with browser execution. To enable fast, installation-free simulations on the web, COPASI was compiled to WebAssembly (Wasm) [23] using Emscripten [24], and a lightweight JavaScript interface provides convenient access to its functionality.

The remainder of the Web interface was developed using React and can therefore be run from a static web server such as GitHub. This makes deployment much easier and doesn’t require an active server to operate. Hosting the web version is simply a matter of moving the JavaScript and Wasm files to a new host.

## 2 Modeling Standards

The Iridium platform uses a variety of community standards. This makes it possible to exchange models and modeling experiments with other software platforms. We define the standards we use in Table 1.

Both the desktop and web platforms support the import and export of SBML. The desktop version also supports the export of SED-ML and COMBINE archives. While SBML is used to describe the model, SED-ML is used to specify the simulation experiments done by the user. This helps to ensure that a given simulation experiment is reproducible. Several tools support SED-ML, such as COPASI, Tellurium, and VCell, as well as non-SBML tools such as OpenCor [27].

To implement SED-ML support, we developed a simple experiment description specification format that can be embedded in a comment section. This structure can be converted to SED-ML. A more detailed description of this format is given in the appendix together with a sample script. Full documentation for this and Antimony is provided as markdown files that can be viewed from the application.

## 3 Comparison to other tools

There are a number of existing web-based and desktop tools available for kinetic modeling, and the availability of a new platform such as Iridium may simply be repeating existing efforts. In this section, we will highlight the unique features of Iridium that make it a worthwhile contribution to the currently available tools. We will only consider GUI-based tools and will exclude scripting-based tools that may use Python, R, or Julia since these are in a completely different space.

We have identified six GUI-based tools currently available to researchers. Historically, there have been more, but we will only consider tools that are still actively maintained and support SBML.

CellCollective [28] is a web-based simulation platform, but currently it only supports Boolean models; we will therefore not consider it in this comparison.

We will split the group into web-based and desktop-focused tools

### 3.1 Web Tools

Of the web-based tools, the most well-known is JWSOnline [29]. This offers an easy-to-use interface with a comprehensive set of analyses. It relies on an active server to operate, in which all calculations are performed using a server-based instantiation of Mathematica [30]. This requires a paid license and an active server, meaning it cannot be run from a GitHub page. JWSOnline offers no slider support and therefore cannot be used to do interactive modeling.

Menelmacar [31] is a new web-based tool from the BioModels team. However, as it currently stands, it can only be used to model recompiled models from the Biomodels repository. In addition, it only offers a limited-time course simulation capability. Users cannot input their own models and interactive modeling is not available.

BNG playground [32] is a new web-based tool that uses a similar approach to the web version of Iridium. For that reason, it is also hosted on GitHub. Models can be described using BioNetGen, which is a specialized domain language for modeling rule-based systems and is more complex than Antimony. The platforms offers a wide variety of analysis, more than the Iridium platform, but the combination of BioNetGen format and a complex interface would make it hard for novices to use. Plotting of results is very slow, but this might be by design. Interactive simulation is limited because a simulation run is only recomputed after the user has finished moving the slider. Therefore, interactive modeling is not possible. However, its range of analyses is impressive.

### 3.2 Desktop Applications

We have identified three desktop GUI applications for comparison.

COPASI [8] is a well-known simulation platform for desktop users. It is comprehensive in its range of analyses and the only one that supports model calibration as part of the tool. Its interactive capabilities are similar to BNG playground, that is, sliders can be invoked, but a simulation is only recomputed when the user terminates the slider movement. To trigger instant recomputation, use track pad swipe gestures, the mouse wheel or the keyboard directional keys.

CellDesigner [33] is also a well-known GUI platform. Its main specialty is being able to render SBGN-like [34] networks. It can run time-course simulations, but the interface is limited, not easy to use, and lacks interactive slider support. It also only supports up to Level 2 SBML, when most published models are at Level 3.

VCell [35] is another well-known modeling tool. This is an advanced SBML-compliant platform focused on spatial modeling but also supports non-spatial modeling; however, it is not an interactive platform.

#### Key Innovations

Given that researchers already have a variety of simulation tools at their disposal, we highlight what we consider the most novel or innovative aspects of the Iridium platform.

The most important innovation is its capability to support true interactive modeling via sliders that display simulation results in real time. Based on our research of current simulators, no simulator has this capability. Coupling a modern interface design with high-performance simulators and modern computer hardware enables a high degree of interactivity. This proved instrumental in our primary use case, which we discuss in the next section. Secondly, users can preserve their simulation experiments either by exporting Python scripts or SED-ML/COMBINE archive files. Thirdly, the BioModels repository is instantly available via a fast caching system with automatic conversion from SBML to the antimony format. The desktop version also includes a novel model checker to identify model errors. For the Web version, we also integrated AI agents that users can use to make queries about models and modeling. These and other novelties are listed in Table 2.

## 4 Use Case: Building a Large EGFR Network

The unique features of the desktop Iridium application are designed to help in the construction of large-scale models, such as those of the EGFR signaling network. Signaling through the EGFR is mediated through the sequential phosphorylation of downstream substrates. The response of the system can be measured experimentally using quantitation phosphorylation and modeled using mass action-based ODEs. However, parameterizing EGFR network models can be very difficult because of both their size and complexity. For example, our current model that includes only the core kinase signaling pathway between the EGFR and the downstream kinase ERK contains 65 reactions, 58 species and 82 parameters. If the initial parameter estimates are not relatively close to the final values, convergence of parameter estimation runs can be difficult to achieve.

The time needed for convergence to optimal values is highly influenced but how close the initial parameter estimates are to them. We addressed the initial parameter estimation problem by overlaying experimental data on a model segment describing that data and then using the sliders to adjust the appropriate parameters. Regulatory networks, such as the EGFR, will show a characteristic time profile in response to a perturbation. They will also show a characteristic dose-response profile. Thus, experimental data used to parameterize the model included both a time series and a dose series.

To usefully combine data and models, it is essential that the data be normalized to the units used for modeling. In the case of the EGFR network model, these are explicitly laid out in the antimony code:


// Unit definitions: 
unit substance = 1e-9 mole; 
unit time_unit = second; 
unit nM = 1e-9 mole / litre; 
unit per_nM = litre / 1e-9 mole; 
unit per_sec = 1 / second; 
unit nM_per_s = 1e-9 mole / (litre * second);
unit per_nMs = litre / (1e-9 mole * second);


The experimental data was normalized to these units by calibrating them against physical standards [36]. The time series data and dose series data contained a common time/dose measurement to facilitate cross-experiment normalization. This improves initial parameter estimates by ensuring quantitative data consistency across different simulation modes.

We use the binding, activation and internalization of the EGFR as an example of how data can be used to generate initial model parameters. The model is a simplified version of the Resat et al model in which the binding of EGF to its receptor both activates the receptor, leading to its self-phosphorylation and accelerated endocytosis [37]. Directly measured parameters include endocytic rates of the activated receptor, dephosphorylation rates of phosphor-EGFR and receptor abundance. Parameters needed to be inferred for the model include the effective forward and reverse rate constant for EGF binding to its receptor and the EGFR self-phosphorylation rate.

To generate date to calibrate the model, EGF at 0.17nM was applied to cells and the phosphorylation level of the EGFR was assessed at 0, 120, 240, 480 and 720 s using mass spectrometry [36]. A dose series in which cells were treated with 0.005nM – 17nM EGF for 240 s was also used. The common point 0.17nM @ 240 s was used as an anchor to normalize the two datasets. The levels of phosphorylated EGFR at each experimental point were quantified and normalized and saved in two text files – one for the time series and one for the dose series.

The controls for loading the data overlay are found below the graph itself (Figure 5). The file should be in standard CVS format with the first row being the labels of the data columns. The title of the first column should be the same as the scanned parameter: “time” in the case of time-series file and “ligand” in the case of the dose series. The color of the default symbols will automatically match that of the simulation result if the labels are the same. Symbols, lines and colors as well as choice of linear or logarithmic graph scaling can be modified using the “Edit Graph” button.

The initial EGFR model was constrained by the measured parameters (ke, $k_{p1}$, Vr, kt). Literature values were used for kf, $k_{p1}$ and kr. As shown in Figure 5, correspondence between the simulated and experimental time series results was very poor. Scanning ligand concentration in the Parameter Scan mode showed similarly poor correspondence (Figure 6, top panels). We then used the sliders to interactively adjust the values of kf, kp1 and kr, initially in the Time Course Simulation mode and then in the Parameter Scan mode. Importantly, the modified parameters were retained when switching modes, allowing a single set of parameters to be optimized using both time-series and dose-series data.

The model with the adjusted parameters displayed good correspondence with the experimental data (Figure 5, bottom panels). The slider values were then copied to the model using the button at the bottom left corner of the Time Course Simulation window in preparation for using a parameter estimation program to refine the final model values.

## 5 Future Enhancements

The current version provides the most basic functionality required of a simulator. However, some key functions are missing. Most noticeable is the lack of support for stochastic simulations. However, both libroadunner and COPASI support stochastic simulation, so this should not be too difficult to include. We excluded it in this version because our primary use case did not require it. Version 2.0 will also include a rewritten high-performance simulator that can run in the web browser. This will bring the web version up to par with the desktop app’s performance. Some possible enhancements are included in Table 3.

Other enhancements include, most notably, model calibration. However, this is a complex workflow, and we have decided not to include it in the current Iridium platform. Instead, we are developing a parallel application for model calibration.

With the recent publication of the CURE guidelines [38], an obvious starting point for enhancements is to include tools that help users meet CURE compliance. These would include automated or semi-automated annotation of models, better support for computational experiment reproducibility, and model checking [39–41]. As a start we have included model checking in the current release, either via access to an AI agent (Web version) or as a built-in tool in the desktop version.

## 6 Conclusion

This article describes the Iridium Platform for simulating biological systems. We describe two Iridium simulation systems. One runs on the desktop (Windows, MacOS) and the other is a web-based application that can be run from a simple web hosting site such as GitHub or Cloudflare. The Iridium Platform provides features that promote ease-of-use by experimental biologists including: sliders for adjusting parameter values while simultaneously displaying simulation results in real time; overlaying experimental data onto simulation results (see use case above); zero-install distribution; AI chat integration; and generation and export of standalone Python implementations that describe the current simulation experiment to support reproducibility.

Both Iridium Simulators are designed around a common workflow involving Control, Input, and Output panels. Model input is provided by the Antimony language, making it very easy to build and edit models. Standard compliance is provided where possible, specifically SBML, SED-ML, and COMBINE archives. A key feature of both versions is interactive simulation, which is one of the primary distinguishing factors of the platform.

## Figures and Tables

**Fig 1.**
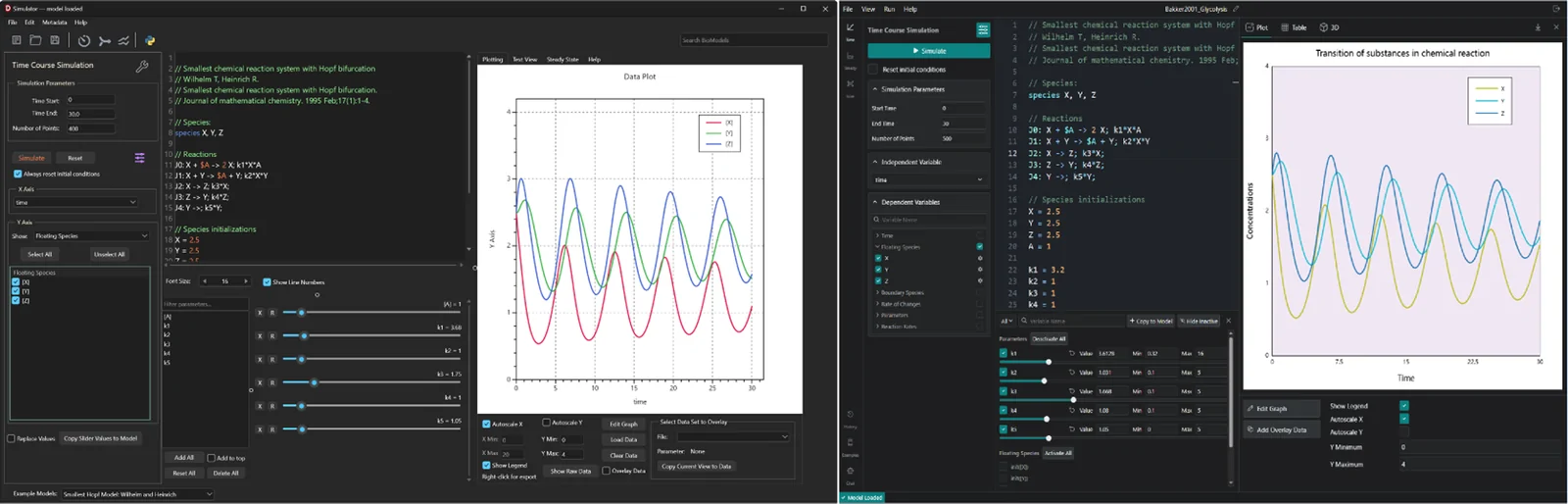
Screenshots of the main window of the desktop version (left) and the Web version (right) of Iridium. To the left is the control panel, the center includes the models and other input features such as the sliders, and on the right is the output panel, which shows a 2D plot of a simulation.

**Fig 2.**
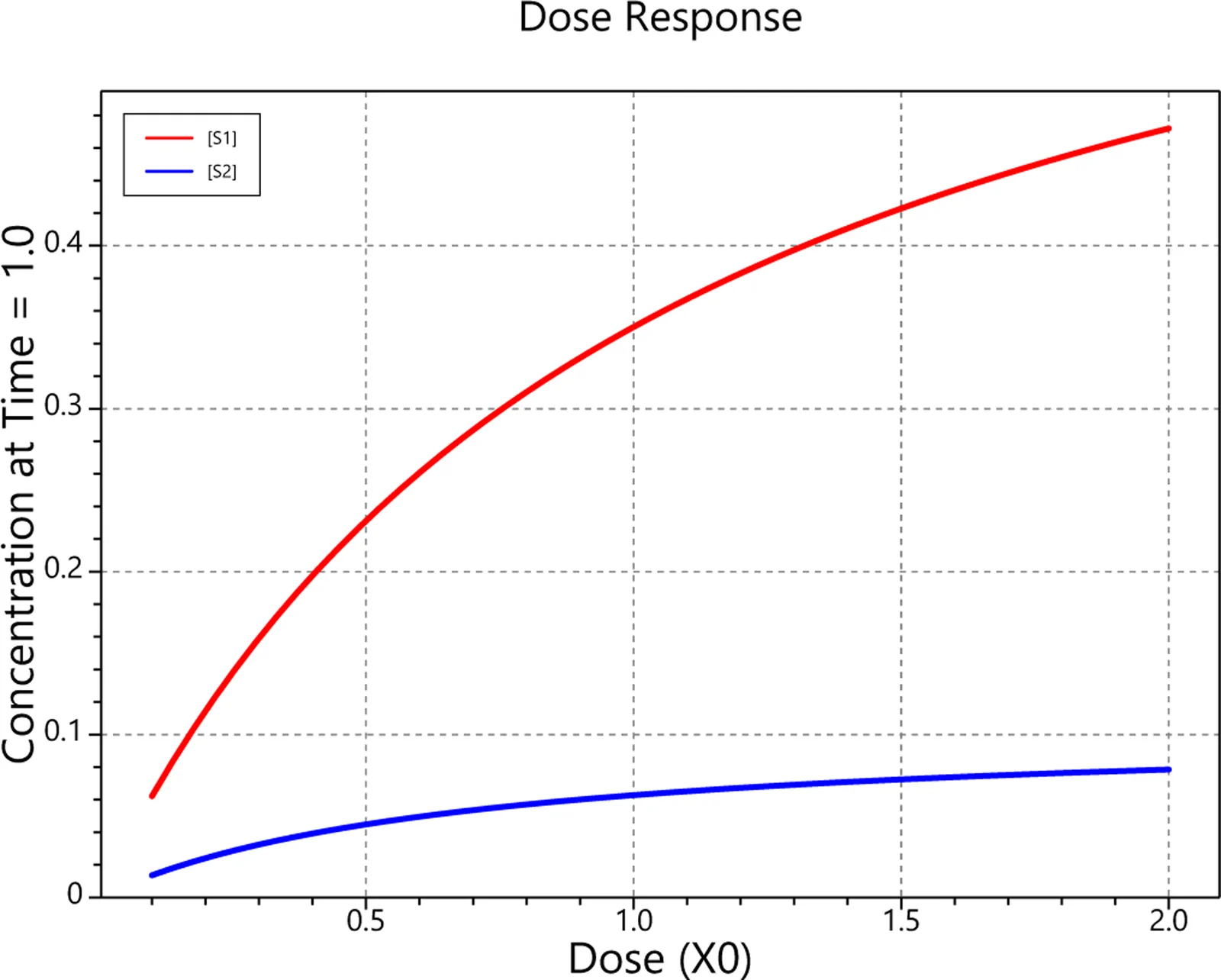
Parameter scan used to trace the effect of dosing the input species, X0 and observing the levels of S1 and S2 at time equal to 1.0. The model comprised three steps X0 -> S1 -> S2 ->, where X0 was a boundary species, with each step governed by a reversible Michaelis-Menten rate law.

**Fig 3.**
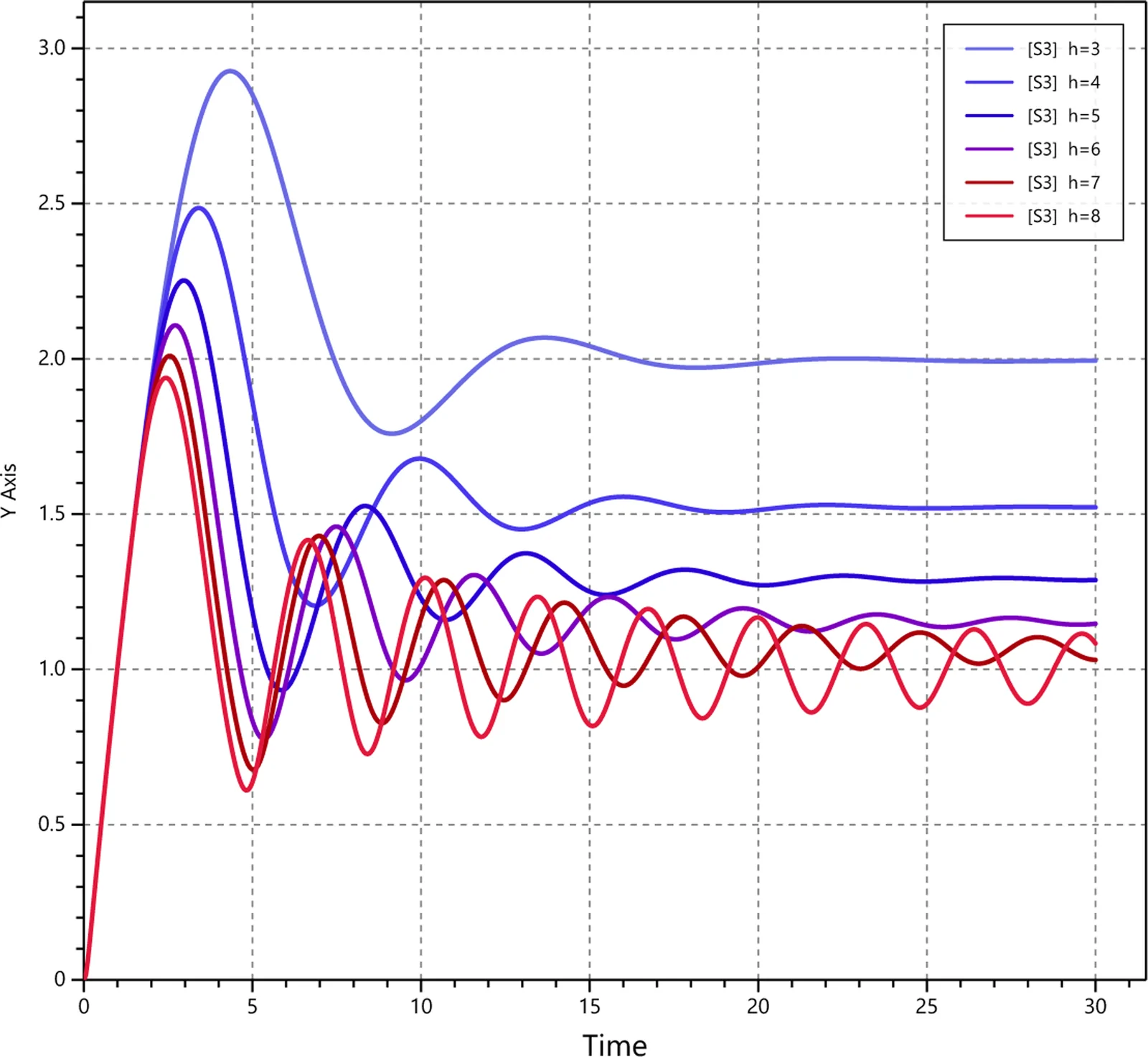
Parameter scan for an oscillating system. The scan changes the model’s Hill coefficient from 4 to 12 across six values. The scan illustrates the onset of oscillations as the feedback strength increases. The exported Python script is shown for illustration purposes in the Appendix.

**Fig 4.**
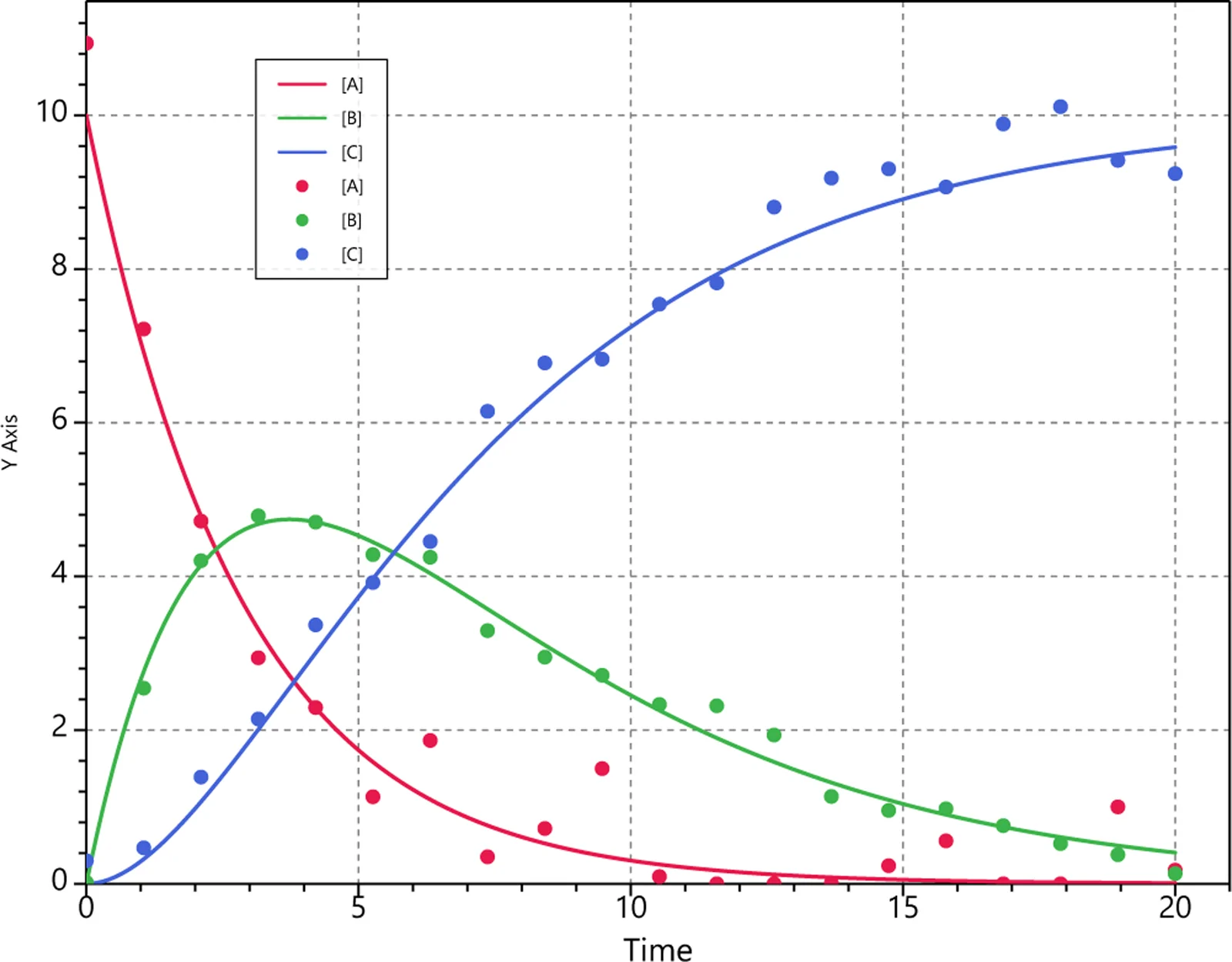
Simulation showing data overlaid onto the results of the simulation.

**Fig 5.**
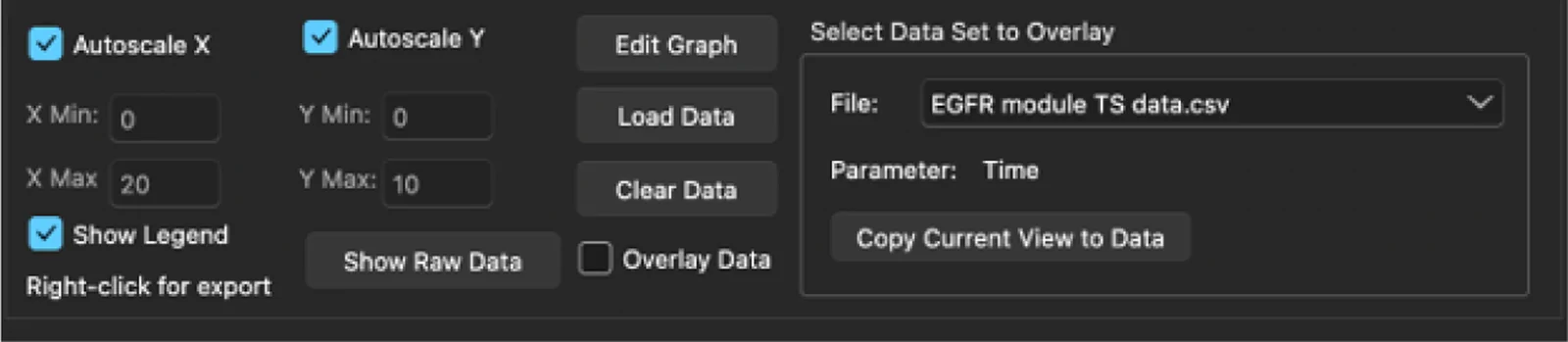
Data and graph formatting controls are located below the main graph window. Multiple files can be loaded and switched by the user, but by default the files are associated with the mode in which they were loaded.

**Fig 6.**
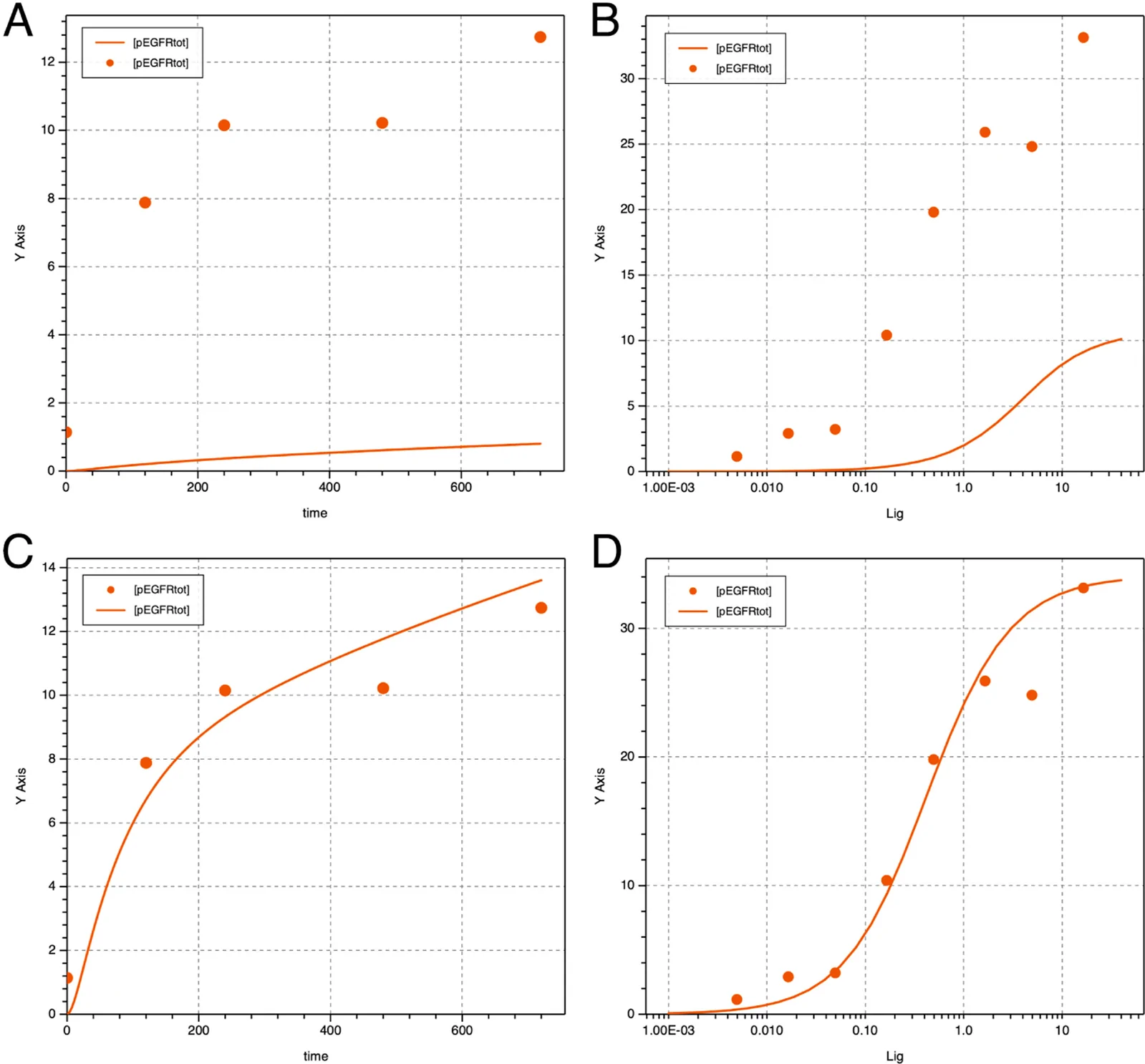
Model simulation with data overlay. A: Time Course simulation of the simple EGFR model overlayed with calibrated experimental data. Non-measured initial parameters were taken from the literature. B: Parameter Scan using “ligand” as a scanned boundary species overlaid with calibrated dose-series data using a log scale. Same parameters as in A. C: same as A using parameters interactively set with the sliders. D: same as B using the same adjusted parameters as C.

**Table 1.** Modeling Standards used by the Iridium Platform

Standard	Purpose
SBML [6]	Systems Biology Markup Language: This is a commonly used standard for exchanging systems biology models between simulation tools or for storing models in repositories such as BioModels.
SED-ML [25]	Simulation Experiment Description Markup Language: This is a proposed standard for describing simulation experiments.
COMBINE Archive [26]	This is a zip file with a manifest that can be used to store the various components of a modeling project. For example, it can store the SBML model as well as the SED-ML file.

**Table 2.** Key Features of the Iridium Platform.

Feature	Platform	Comment
Reproducibility	D, W	Export of SBML/Antimony
Reproducibility	D	Export of Python scripts, SED-ML and COMBINE archives
AI Support	W	The Web version has an embedded AI window that can be used alongside the modeling study.
Single window interface	D, W	A single structured window interface makes learning the platform much easier.
BioModels interface	D, W	Both versions has fast cached access to the BioModels repository with automatic translation to Antimony syntax.
Export Plots as PDF	D, W	Export simulation results in publication-ready DPF files.
Data Overlay	D, W	Ability to overlay experimental results onto simulation results.
Model Checker	D, W	The Web version has an AI interface that can be used to check for model errors, while the Desktop version has a dedicated model checker built in.
Software Innovations:		Web: Zero install, reduced maintenance requirements Desktop: AI-assisted development with future maintenance and new feature additions.

D = Desktop version; W = Web Version

**Table 3.** Potential enhancements for version 2.0

Enhancement	Comments
Enzyme kinetics rate laws support.	Remembering specific enzyme kinetic laws is difficult. This enhancement can provide a database of possible enzyme laws that the user can choose from.
Stochastic Simulation	Augment deterministic modeling based on differential equations with stochastic simulation.
Better SED-ML/SED2 support	Enhance support for computational experiment reproducibility.

## Data Availability

The desktop software is available as binary executables for Windows and Mac OS. The source code is open source, licensed under the Apache 2.0 license, and available at https://github.com/sys-bio/IridiumSimulator. The Web version is available via a static GitHub page and runs on all platforms. The code is open source and licensed under the Apache 2.0 license and is available at https://github.com/sys-bio/WebIridium

## Appendix

### Python Script Generated for Figure 3

# Python script generated by Iridium parameter scan. 
# Reproduces the scan using Tellurium.

import tellurium as te 
import matplotlib.pyplot as plt 
import numpy as np

# ----Model ----
r = te.loada(r"""

// A negative-feedback oscillator
// Originally from a model by Athel Cornish-Bowden

// Reactions:
J0: $X0 => S1; VM1*(X0 - S1/Keq1)/(1 + X0 + S1 + S4ĥ)
J1: S1 => S2; (10*S1 – 2*S2)/(1 + S1 + S2)
J2: S2 => S3; (10*S2 – 2*S3)/(1 + S2 + S3)
J3: S3 => S4; (10*S3 – 2*S4)/(1 + S3 + S4)
J4: S4 => $X1; V4*S4/(KS4 + S4)

// Species initializations:
S1 = 0; S2 = 0; S3 = 0
S4 = 0; X0 = 10; X1 = 0

// Variable initializations:
VM1 = 10; Keq1 = 10 
h = 10; V4 = 2.5; KS4 = 0.5
""")

# ---- Selection ----
selection = [’time’, ’[S3]’]

# ---- course settings ----
time_start = 0.0 
time_end = 20.0 
num_points = 500

# ---- Scan parameter and range ----
scan_param = ’h’ 
scan_values = np.linspace(2, 12, 6)

# Colors below match the on-screen plot exactly, 
# read from the live series. Edit to restyle. 
colors = [’#0000FF’, ’#1500EA’, ’#2A00D5’, ’#3F00C0’, ’#5500AA’, ’#6A0095’]

# ---- Scan ----
color_idx = 0 
for k in scan_values:
      r.reset()
      r.setValue(scan_param, k) 
      m = r.simulate(time_start, time_end, num_points, selection) 
      for j in range(1, len(selection)):
	  c = colors[color_idx] if colors else None 
	  plt.plot(m[:, 0], m[:, j], color=c, 
		   label=f’{selection[j]}, {scan_param}={k:.3g}’)
	  color_idx += 1
plt.legend() 
plt.show()

### Sample Report from Model Checker

#### Model Check Report

2 reaction(s) against 18 rate law(s), structure and behavior.

### What was matched

**Table:**

Reaction	Rate law	Why
_J0	michaelis_menten_irrev	a close structural match, distance 0.429
_J1	mass_action_irrev	an exact structural match

Distance is how far a reaction’s expression sits from the law it was matched to: 0.000 is an identical shape, 1.000 nothing in common.

### Findings

#### J0 (line 9)

**D004 ERROR** (checked against michaelis_menten_irrev)

this law requires the rate to approach 0.01 as S runs to inf, but at S = 1E010 it is 1E010 and is not closing on it

- seen at: Km=0.01, Vm=0.01; S=1E010 gives 1E010, expected ~0.01

**D005 ERROR** (checked against michaelis_menten_irrev)

this law requires the rate to be 0.005 at S=0.01, but it is 0.01

- seen at: Km=0.01, S=0.01, Vm=0.01; rate=0.01, expected 0.005

#### D101 ERROR

this reaction divides by zero at the values this model starts with (Km = 0), so it cannot be simulated from time zero

- found: k1*A/Km
- seen at: k1=0.35, Km=0, A=10

**S010 ERROR** (checked against michaelis_menten_irrev)

the right quantities, but bracketed differently from this law

- found: k1*A/Km
- expected: Vm * S / (Km + S)
- suggestion: the law reads Vm * S / (Km + S)

**D006 WARN** (checked against michaelis_menten_irrev)

this differs from the law by up to 100.0% over the sampled range

- expected: Vm * S / (Km + S)
- seen at: Km=0.01, S=1000, Vm=4.64159; rate 464159 against 4.64154, a 100.0% difference

**S011 WARN** (checked against michaelis_menten_irrev)

“k1” plays the Vm role, which is conventionally called Vm or Vmax or V_max or vmax or Vf

- found: k1
- expected: Vm

#### J1 (line 10)

##### S018 INFO

“B” has no initial value, so it starts at zero

- found: B

**4 errors, 2 warnings.

### Simulation Description Format

To give users the ability to describe a simulation experiment, we implemented a simple comment-based metadata format. This format is compatible with SED-ML, thus allowing users to share their simulation experiment with other users.

Simulation metadata is written inside Antimony block comments, so an annotated model remains a valid Antimony file and is ignored by tools that do not recognize the notation. Within a block comment, a command region begins at any line whose first non-whitespace character is @ followed by a command name, and extends to the end of that command’s value; text outside a command region is treated as prose and ignored, allowing a descriptive comment and its associated commands to occupy the same block. A command consists of the @ character, a command name, an optional user-supplied label, a colon, and a series of key: value statements enclosed in braces. Values may be numbers, quoted strings, identifiers, booleans, hexadecimal colors, bracketed lists, or nested objects. Whitespace, including newlines, is ignored. key: value pairs are comma-separated. User comments in the form // can still be used to add comments to block. The notation provides no expressions, variables, or control flow. Commands may appear in any number of comment blocks and are processed in file order as though concatenated.

Six commands are defined: @meta records title, author and description; @simulate and @steadystate specify time-course and steady-state tasks; @scan repeats a task over a range or explicit list of values of one parameter, reducing each run to a stated measure; and @plot and @output describe figures and tabular output derived from those tasks. Tasks carry optional labels and can be chained by a source key, so a figure states which run, for example, a simulation will use. A set object on any task assigns values to model quantities such as parameters, species initial values, or compartment sizes, for the duration of that task alone. The parsed representation can be exported as a roadrunner/Tellurium Python script or as SED-ML (Level 1 Versions 3 and 4), optionally packaged as a COMBINE archive that also includes the model. Full documentation for this format is provided as markdown files available within the desktop application itself. The following is a sample file that specifies a time course simulation followed by a plot.

$Xo -> S1; Vmax * Km / (Km + S1)
  S1 -> S2; 0.3 * S1
  S2 -> ; 0.2 * S2

S1 = 1.0; S2 = 0.5
Vmax = 1.0; Km = 0.5

/*
  @simulate: { timeend: 50, points: 500 }
  @plot: { 
    y: [S1, S2], 
    title: "Wild type",
  }
*/

### Use of AI

This project inadvertently served as a use case for using AI to assist in developing sophisticated applications. In this section, we will describe our experience.

The desktop and web editions of Iridium both use modern AI technology but in different ways. The Web version has a dedicated AI panel where users can ask questions about modeling or even assist in building a model. Currently, it supports Claude and ChatGPT access.

The current desktop version was built entirely using Claude Code [42]. An earlier version of the desktop application exists, and was developed using traditional manual coding techniques. The use of AI to write teh second version resulted in a number of key ad vantages: 1) Development time was much faster, weeks rather than months; 2) Maintenance and bug resolution are now much simpler and handled by AI conversations; 3) Three new GUI skia components were written from scratch by Claude Code to ensure the functionality was available on the Mac OS. These included the plotting panel, a Markdown viewer, and the syntax highlighting editor.

### Lessons Learned and Observations on the use of AI

#### Specification Document:

A useful lesson learned in using AI for coding was the value of first creating a specification document before attempting to write code. This offered a number of advantages:

1. It forces a human developer or user to sketch out the basic features required in the application. This allows the AI to propose architectural designs before coding starts.
2. It helps the AI determine the order of construction and it sets up a series of milestones it can follow. This also helps the AI avoid design errors early in the process, resulting in significantly fewer code changes later on. For example, in designing the editor, a redo/undo system is easier to integrate in a later milestone if the AI ‘knows’ that this is a future feature to add and prepare the ground early on;
3. A specification document allows the AI to plan a more rigorous testing regime for each milestone and will do regression testing on all earlier milestones where applicable. The AI will offload backend logic or algorithms for testing to separate console-based test harnesses. This enables the AI to test specific algorithms without invoking the full GUI application.
4. A specification document can be handed to another developer group, which they can use it to implement the same application in another framework, such as C++ or Rust or even a web-based application.

For any large project, we therefore recommend creating a specification document first. This practice is well documented by many developers and companies [43] and contrasts with pure ‘vibe programming ’, which relies on casual, conversational AI prompting without any planning. In practice, specification-driven coding is more of a hybrid because the specification document is a living document and changes as unanticipated technical constraints emerge or user requirements evolve during implementation. The most important aspect of specification-driven coding is not rigidity but enforced testing and a document that the AI can follow and use to plan with. It also encourages the AI to go into agentic mode, which can result in more robust software.

#### Human Guidance:

While an AI can autonomously write the code without human intervention, the initial architectural design, user requirements, and monitoring of the AI are essential. An AI can sometimes go down ‘rabbit holes’ and needs human intervention to escape and reassess. Often when this happens, it is advisable to start a new session so that it ‘forgets’ the error it just made but can also be told not to follow this type of design in its configuration file.

#### Testing:

The greatest impact on the performance of code written by an AI is by using an agentic tool like Claude Code. This gives the AI the opportunity to obtain as much feedback as possible on how the code performs. This can involve timing code runs and assessing code efficiency, running numerous tests to ensure individual features or subroutines function as expected. For GUI applications, the AI will also, of its own volition, control a GUI application from the keyboard and mouse and take screenshots to assess that the GUI is performing as expected. For applications that require algorithm development, the AI will create its own separate test harness to ensure the algorithm functions correctly before integrating it into the main application. Each harness gets its own set of tests. An agentic AI will carry out its own testing and bug fixing, which greatly reduces the chance of code ‘hallucinations’.

#### GUI Development:

Claude Code has proved to be very good at creating graphical user interfaces. For example, the parameter scan panel was designed entirely by the AI, based on prompts from the human developer. However, it is not perfect and required some adjustments. Delphi has a visual designer, so final polishing of the interface is straightforward.

The final desktop application amounted to just over 98,000 lines of code. Multiple sessions were required for this level of effort because Claude’s context window is limited to 1,000,000 tokens. However, Claude Code keeps a record of all its past dealings in the Claude.md file it maintains. A key advantage of using AI has been the reduced cost of maintenance and adding new features, which is now much easier.

### Software Technology

#### Model Representation

Models are represented to the user in the Antimony format [44, 45] with syntax highlighting. This is an easy-to-use format for describing biochemical networks. The current version of Antimony supports SBML Core, distribution packages, and layout and rendering. Text input is via a straightforward editor that is both familiar and arguably faster than inputting a model via a spreadsheet. It is also much faster than using a dedicated GUI that has buttons to add, change, or delete species and reactions or even using a Python-based model construction APIs, such as PySB [46] or Basico [47].

#### 7.1 GUI Technology

Today, many choices exist for developing graphical user interfaces for desktop applications. Almost all rely on hand-crafting the user interface programmatically. This can make the design of sophisticated GUIs time-consuming and costly. We have experimented with wxPython [48, 49], WinForms [50], and QT [51] frameworks for designing GUIs. Python-based GUIs that use wxPython can be slow due to the underlying Python interpreter. QT-based apps are a realistic alternative, but the design-side engineering is non-trivial. WinForms is another possibility, but Microsoft has not been fully committed to it and keeps developing new frameworks such as WPF, UWP, and WinUI 3, which are not cross-platform. A summary of the pros and cons of a number of GUI frameworks can be found at RoyalSloth [52]. Although dated 2020, it paints a fairly negative picture of GUI development frameworks.

In 1991, Microsoft released Visual Basic, which provided a completely new way to build GUI applications based on a drag-and-drop approach (itself based on on Alan Cooper’s Ruby). This allowed complex GUI interfaces to be rapidly built. However, Visual Basic was built on the BASIC language, which used an underlying interpreter. This made software written in Visual Basic less performant. Nevertheless, several software companies started to copy the approach. Probably the most famous of these was Borland, which was the author of the very successful Turbo series. In 1995, Borland released a successor to Turbo Pascal, called Delphi. This used the same visual approach that was used by Visual Basic, but the underlying language was natively compiled. Thus, the performance issues were resolved. Although the underlying language was Pascal, it was a greatly enhanced version called Object Pascal, which included a sophisticated object model as well as pioneering modern exception handling and object properties. Since 1995, 38 editions of Delphi have been released, with the current edition (Florence, 13.2) released in 2026. In addition, the language has been continuously enhanced and now includes features such as interfaces, anonymous methods, generics, runtime type information etc. What stands out, however, is the GUI support. Since 2011, GUI support has been cross-platform for Windows, Mac, Linux, and mobile devices. This allows the same code base to be used for all platforms. This is particularly advantageous for desktop applications, which greatly reduces costs in having to maintain multiple code bases.

The emergence of large language models has, however, transformed the development of software and frameworks such as Delphi, are ideally suited to LLM based design because their design specifications use plain text (DFM files), which is ideal for LLM consumption and generation. The GUI for Iridium was built using FMX, Delphi, and Skia [53]. The code can be compiled using the free Community Edition of Delphi, version 13.0.
